# Welcoming couples into the Assisted Human Reproduction Program: developing educational technology based on the context*

**DOI:** 10.1590/1980-220X-REEUSP-2024-0263en

**Published:** 2025-07-07

**Authors:** Alzinei Simor, Ivonete Vieira Pereira Peixoto, Mônica Custódia de Couto Abreu Pamplona, Marcia Helena Machado Nascimento, Fernando Conceição de Lima, Rubenilson Caldas Valois, Mary Elizabeth de Santana, Elizabeth Teixeira

**Affiliations:** 1 Universidade do Estado do Pará, Belém, PA, Brazil.

**Keywords:** Reproduction, Infertility, Nursing, User Embracement, Educational Technology

## Abstract

**Objective:**

To describe the process of developing an educational technology for nurses with a view to mediating the welcoming of couples in assisted human reproduction.

**Method:**

Development research with a methodological interface, developed in two phases: situational diagnosis in the context; and product development. Diagnosis was conducted through semi-structured interviews with 20 couples, in Belém, Pará, Brazil, from June to September 2019, and the IRAMUTEQ® software was used to support data analysis. Production used textual and image resources and the Corel DRAW GraphicsSuite X6 program.

**Results:**

From the *corpus*, six classes emerged, which were organized into three categories representative of the context. From what emerged, an educational technology in the form of a folder was produced to mediate the welcoming of couples by a nurse.

**Final considerations:**

Couple conditions indicated the need for informed support and the survey, and the application of social evidence favored the development of an educational technology based on the context.

## INTRODUCTION

Infertility is clinically defined as the failure to achieve pregnancy after 12 months or more of regular unprotected sexual intercourse. It is a growing public health problem worldwide, with a lifetime prevalence of between 17% and 26%^(1)^. It should also be noted that the chance of a fertile couple becoming pregnant is approximately 15% to 25% per month, and after a year of trying, this cumulative rate will be approximately 80%. Therefore, appropriate medical interventions and an investigation into infertility are necessary^(2)^.

It is imperative that, for many couples, the desire to become pregnant is a necessity, but one that comes up against important health conditions. Pregnancy and motherhood are health issues and should, therefore, be ensured through public policies that guarantee full access to services for infertility treatment and/or assisted human reproduction (AHR), overcoming access barriers as a matter of reproductive justice^(3)^.

In this scenario, nurses play a crucial role in care comprehensiveness in the Assisted Human Reproduction and Fertility Program, providing support, welcoming, clarifying medical instructions and offering educational guidance, specifically during welcoming, with special attention to patient education, focusing on physical and psychological care, guidance on procedures, promoting quality of healthcare and sensitivity to the needs and stories of couples/individuals^(4)^.

With the progress of the AHR area in recent times, nursing has gained prominence in this scenario, fundamentally ensuring humanized welcoming, in addition to acting as the main articulator of information, conduct with the multidisciplinary team and in the AHR program team coordination, promoting assistance targeted to couples’ needs, guiding and preparing them in all procedures as part of welcoming^(5)^.

In the state of Pará, the infertility care service provided by the Brazilian Health System (In Portuguese, *Sistema* Único *de Saúde* - SUS) is provided at the Maternal and Child Reference Unit (In Portuguese, *Unidade de Referência Materno Infantil* - UREMIA), which welcomes infertile couples of all social classes and genders, trying to become pregnant through an assisted reproduction program. In this service, educational tools, such as educational technologies (ETs), are not used during the welcoming of infertile couples. Thus, it is believed that the link between ET and medicalization and humanization/welcoming values nurses’ work in relation to reproductive biotechnologies^(5,6)^.

It is known that ET, as a tool, can mediate the planning, implementation and assessment of the teaching-learning process used in educational meetings, strengthening the construction of knowledge between student and educator^(7)^. As strategies for health education, ETs contribute to increasing access to information regarding illness, diagnosis, treatment and access to healthcare services, considering the social and family context^(8)^. ETs can be considered a significant educational and care resource, and can be used in health education activities^(7)^.

Therefore, it is believed that investing in the welcoming and production of ET for this clientele can reduce the fears, anxiety and impact of couples in relation to the “desire to become pregnant”, as well as clarify doubts regarding reproductive health, necessary tests, and the situation and functioning of the Assisted Human Reproduction Program^(9)^.

Thus, this study aimed to describe the process of developing an educational technology for nurses with a view to mediating the welcoming of couples in AHR.

## METHOD

### Study Design

This is development research^(9)^ with methodological interface^(10)^, developed in two phases. The first phase (situational diagnosis in the context of AHR) was research with a qualitative approach, according to COnsolidated criteria for REporting Qualitative research recommendations. The second phase (product development)^(9)^ was based on the context^(11)^. The context-based production method consisted of collecting information/considerations about the target audience and with the target audience (couples served in the AHR program), obtained through primary data production, making it possible to identify significant topics and, from them, develop ETs^(11)^. It should be noted that this study has not yet been made available on the service and participants have not yet been able to access the final version; however, it will go through the stages of continuity projects^(12)^, with subsequent assessment with the target audience in future studies.

Phase I - Situational diagnosis in the context of assisted human reproduction

Place

The study was developed in a UREMIA, a state reference in AHR, linked to the SUS, located in the city of Belém, Pará, Brazil.

### Population and Selection Criteria

Twenty infertile couples who sought treatment for AHR at UREMIA participated in the study. Inclusion criteria were couples aged between 18 and 40 years, who had been followed up at the referral unit for at least six months. Exclusion criterion was couples in which a woman had undergone a hysterectomy and/or had an underlying disease under treatment (uterine fibroids, endometriosis). Participants were approached in the waiting room, before the consultation with a nurse, and at that time, they were invited to participate in the study, and those who accepted were taken to a private room where they were welcomed by the researcher and informed about the procedures to be performed.

### Sample Definition and Data Collection

Data were collected between June and September 2019. Semi-structured interviews were conducted using a script constructed with 22 questions about couples’ sociodemographic profile and conditions. The questions were related to: couples’ knowledge about the Assisted Human Reproduction Program; reasons for seeking care in the program; means and ways for the program to help couples in welcoming; suggestions for meaningful content to be shared in an ET; and suggestions for an interesting ET format to gather content with a view to helping infertile couples receiving care in the program.

### Data Analysis and Processing

The interviews were transcribed by the researchers and organized into a text *corpus*. Subsequently, the *corpus* was analyzed using the three stages of thematic content analysis, with the support of *Interface de R pour les Analyses Multidimensionnelles de Textes et de Questionnaires* (IRAMUTEQ^®^) software, according to the Reinet method^(13)^.

IRAMUTEQ^®^ enables the identification of context in which words occur, performing a lexical analysis of the textual material and dividing the text into hierarchical classes, which are identified from text segments (TSs) that share the same vocabulary, thus making it easier for the researcher to understand their content. The class is defined as a grouping made up of several TSs with homogeneous vocabulary^(14)^.

The Descending Hierarchical Classification (DHC) method was chosen, in which texts were classified according to their respective vocabularies, and the set of them was divided by the frequency of reduced forms. The focus was on acquisition of TS classes that presented similar vocabularies among themselves and different from TSs of other classes. For DHC analysis, it was necessary to have a percentage of use of at least 75% of TSs by IRAMUTEQ^®^, considering that if retention was lower than the proposed one, the *corpus* was not representative for this type of analysis or that the *corpus* content was very diverse, not allowing them to be hierarchized^(14)^. Thus, after processing, the categories generated from DHC classes were subjected to the thematic analysis process.

### Ethical Aspects

This study followed the ethical precepts of research involving human beings, listed in Resolution 466/2012, and was approved by the Research Ethics Committee of a public university in northern Brazil. Participants’ consent was obtained through the Informed Consent Form, under Opinion 3.308.311. To preserve anonymity, participants were given the letters “IC” for infertile couples, followed by the number corresponding to the order in which the interviews were conducted.

Phase II - Development of educational technology on the support of couples in assisted human reproduction

## RESULTS

### Situational Diagnosis

The characteristics of the 20 participants indicated that their ages ranged from 18 to 40 years. Of these, 12 (60%) reported being in a stable union and eight (40%) were married. Regarding their level of education, 14 (70%) had completed high school and six (30%) had incomplete high school. Moreover, 19 (95%) couples reported a family income of one minimum wage and one (5%) couple reported a family income of more than one minimum wage. Concerning religion, 12 (60%) of couples considered themselves Catholic and eight (40%) considered themselves Evangelical.

**Figure 1:**
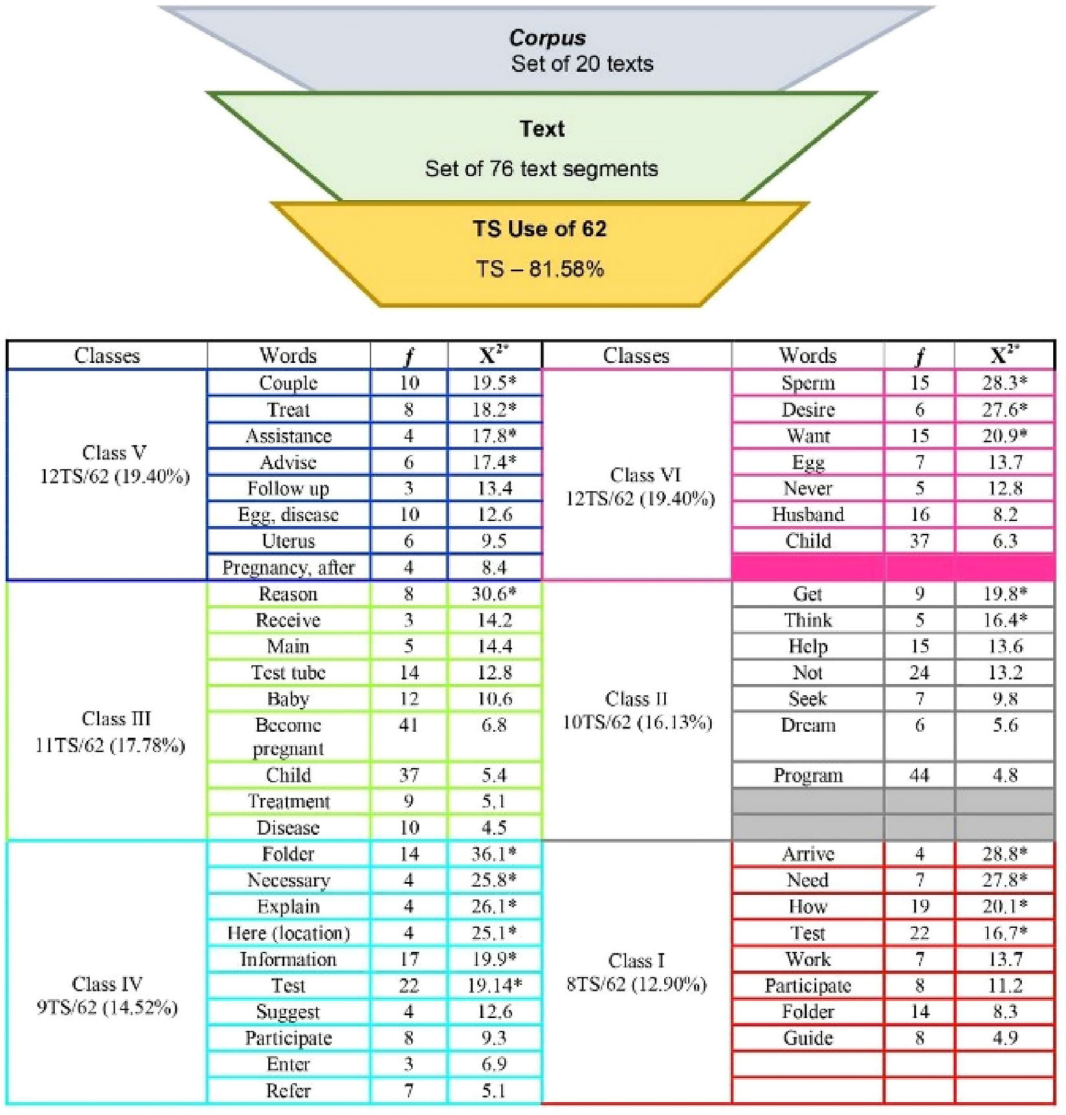
Classes and words with their respective frequencies and chi-square originated from the process of analyzing data from the text corpus “Educational technology for couples in the Assisted Human Reproduction Program”. Belém, Pará, Brazil, 2020.

Note: *Significance level of the word with the class of <0.0001; TS – text segment; f – class frequency; X2 – chi-square value.

The text *corpus* presented 20 text units, 76 TSs, 407 distinct forms and 2,667 occurrences of words in the text, with an average frequency of the forms of 35,092,105, generating six distinct semantic classes. Sixty-two TSs were used out of a total of 76, which corresponded to 81.58% of the *corpus*, characterizing the TSs. The DHC identified the relationship between the classes and the words with the greatest association in these classes and their respective chi-squares and frequencies, having reported only those that met the established criterion [χ^2^ (1) ≥ 3.84, p < 0.05], i.e., the significant words (Figure 1).

**Figure 2:**
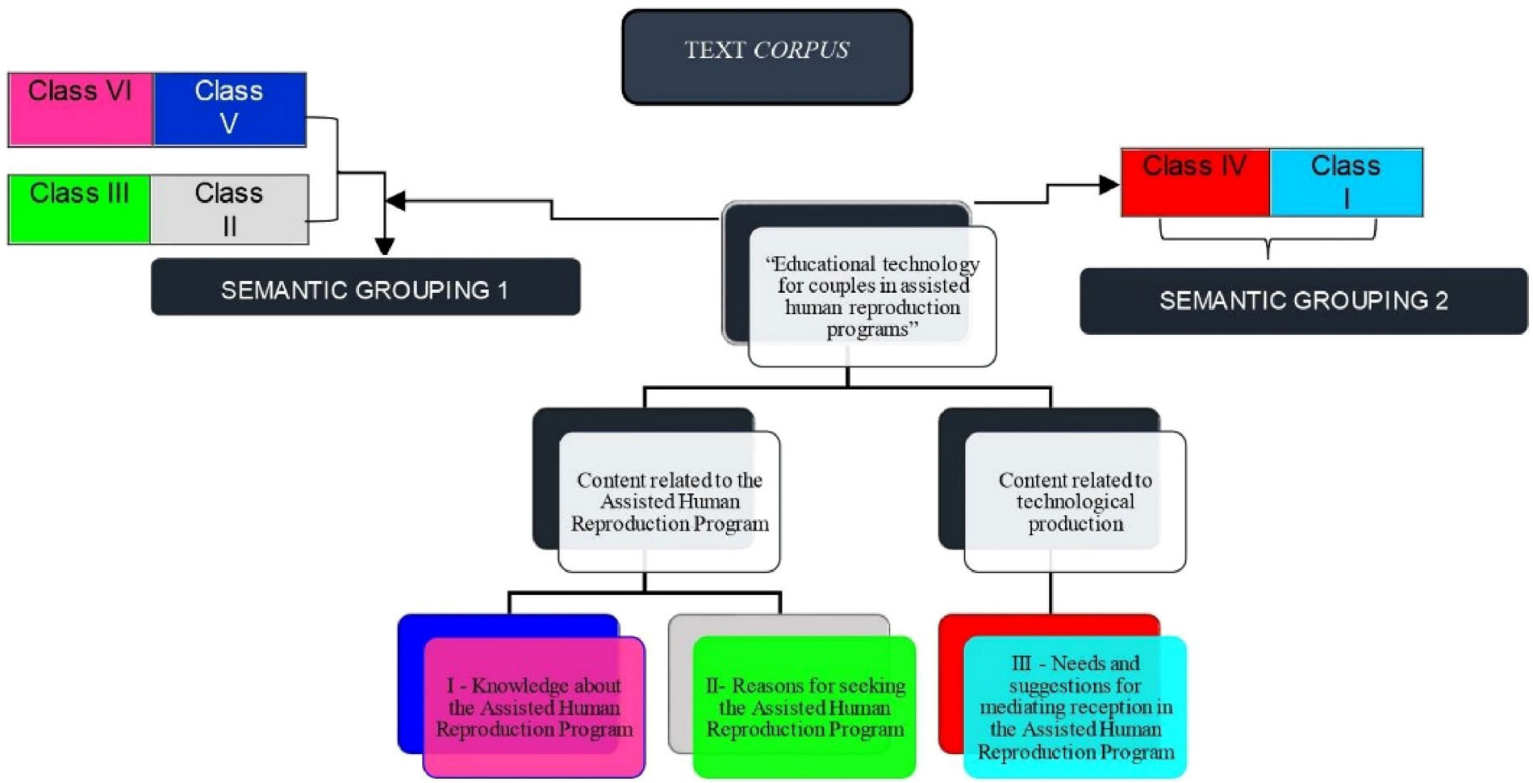
Thematic categories originated from the process of analyzing data from the text corpus “Educational technology for couples in the Assisted Human Reproduction Program”. Belém, Pará, Brazil, 2020.

From this, the thematic categories emerged from semantic group 1, which are contents related to the Assisted Human Reproduction Program, and from semantic group 2, which are contents related to technological production, originating from the six classes generated by IRAMUTEQ^®^, which lexically explored the text *corpus* “Educational technology for couples in the Assisted Human Reproduction Program”. The respective thematic categories were interpreted based on the content analysis proposed by Bardin^(12)^, organized in a flowchart format (Figure 2).

#### Category 1: Knowledge About the Assisted Human Reproduction Program

In this category, in the arrangement of classes VI and V, it was found that the words “sperm” (n = 15) and “couple” (n = 10) were the most prominent and the most specific, with high significance. The word “child” (n = 37), in turn, was the most cited, which encompassed a unique meaning, as it expressed couples’ cognitive association about the Assisted Human Reproduction Program. The words that stood out were “treat”, “assistance”, “advise”, “follow up”, “egg”, “disease”, “uterus”, “pregnancy”, “desire”, “want” and “husband”, revealing the limited knowledge that couples have about the Assisted Human Reproduction Program. The couples, therefore, reported:

*The Assisted Human Reproduction Program provides guidance to couples on how to become pregnant. They can take my egg and my husband’s sperm and implant it in me, and the program monitors the process. We think that’s it!* (IC-01)

*In our opinion, the Assisted Reproduction Program will advise couples who want to become pregnant. It will tell them who can, what is needed, whether they need to do any tests, etc. I think that’s it!* (IC-03)

*The program will combine my egg and my husband’s sperm. I want to become pregnant and have a child, so that when we get older, we will have someone to help us, to find out the difficulty that prevents us from becoming pregnant, and the professionals can solve the difficulty we have in becoming pregnant and treating this disease.* (IC-06)

#### Category II: Reasons for Seeking the Assisted Human Reproduction Program

In category II, classes 2 and 3 merged, and the words “reason” (n = 8) and “get” (n = 9) stood out the most, i.e., they achieved high significance in this class. The words “program” (n = 44), “become pregnant” (n = 41) and “child” (n = 37) were the most cited and were not statistically significant; however, they add relevant meaning to the context of the category, as they represent the main reason that led couples to seek the service, the desire to become pregnant, have a child and discover the cause of the difficulty in becoming pregnant. The couples stated:

*The main reason we sought out the program was to help us become pregnant and have a child, receive guidance on sexual intercourse, treatment to become pregnant and, finally, place an egg and a sperm in my uterus to become pregnant.* (IC-17)

*The main reason is to want to become pregnant and have a child! We want help to have a baby. A healthy child!* (IC-19)

The following words that stood out were “test tube”, “baby”, “treatment”, “disease”, “think”, “help” and “dream”, confirming couples’ reasons for seeking the Assisted Human Reproduction Program services. Thus, they said:

*Fulfilling my dream of carrying our child, giving me the guidance and information I needed to become pregnant.* (IC-06)

*Since they make test tube babies, we want to have one. We don’t have any diseases; I think he can help us become pregnant and have children and find out why it’s difficult to become pregnant.* [...] *the program will watch the conception of a child. I looked for the program to find out the disease that prevents us from having children. We want to become pregnant even if it’s a test tube!* (IC-09)

*The main reason is for me to have a child and become pregnant, because I have never been pregnant! To make this dream come true, I believe the program will treat this disease and examine my eggs and my husband’s sperm.* (IC-15)

#### Category III: Needs and Suggestions for Mediating Welcoming in the Assisted Human Reproduction Program

Category III emerged from the association of words contained in classes 1 and 4, and it was found that the words “folder” (n = 14) and “arrive” (n = 4) stood out the most, being more specific and reaching high significance in this class. The word “test” (n = 22) obtained a degree of significance and was cited in both classes, which encompassed a special meaning by expressing the association of couples’ doubts about the tests necessary to enter the Assisted Human Reproduction Program. The couples, therefore, mentioned:

*Could you have a folder with information like this: what is the program? How do I join? What tests are required? Who can do this?* (IC-04)

*That has information on how to get to the unit, what is needed to be part of it, if tests are needed? What is the program about assisted reproduction? All this in a folder!* (IC-06)

*A folder with more complete information: how does the program work? What is the program? Who can participate? How can I be referred here? What tests are needed?* (IC-12)

The following words that stood out were “necessary”, “explain”, “information”, “participate”, “refer”, “function” and “guide”, revealing couples’ suggestions regarding the content to compose a folder, which was the ET of choice to be developed in this study.

It was inferred that the couples suggested a folder because it was clear and precise, easy to handle and objective to read. It was also clear from the reports that the couples were eager for information about how the Assisted Human Reproduction Program works, as well as the tests, the therapeutic itinerary, the referrals, the admission, among others, as observed in the statements:

*It could be a folder with more information, such as explaining who can and cannot become pregnant, how old you can be to undergo assisted reproduction, and if the person has had a tubal ligation, they can be part of this program. Explain the tests and referrals!* (IC-01).

*More information, such as who can participate, the tests they need, how to get here. These things could all be in a folder* (IC-08).

*We suggest a folder with information about what the program is, how it works, who can participate, how to get here, and whether tests are required. A folder informing who can and cannot participate?* (IC-11).

### Development of Educational Technology

Based on the topics that emerged, the folder format was chosen, considering participants’ interests and the content needed for welcoming. The folder was prepared on two A4 sheets (21 cm x 29.7 cm), divided into three parts, aiming at the possibility of being folded when printed, totaling six pages, in landscape orientation. Calibri and Times New Roman fonts were used, varying in font size, being 12 for the body of the text, 14 for the topics, and 20 for the title. The title “Assisted Human Reproduction Program” contemplated the folder’s objective.

The folder was structured into five topics: “What is the Assisted Human Reproduction Program?”; “Who can participate?”; “How is the referral to the program made?”; “What tests are necessary?”; “How does the Assisted Human Reproduction Program work?”. The main information was made available with an objective demonstration of the expected action, avoiding accumulation of guidelines, with short, simple sentences and use of active voice.

**Figure 3:**
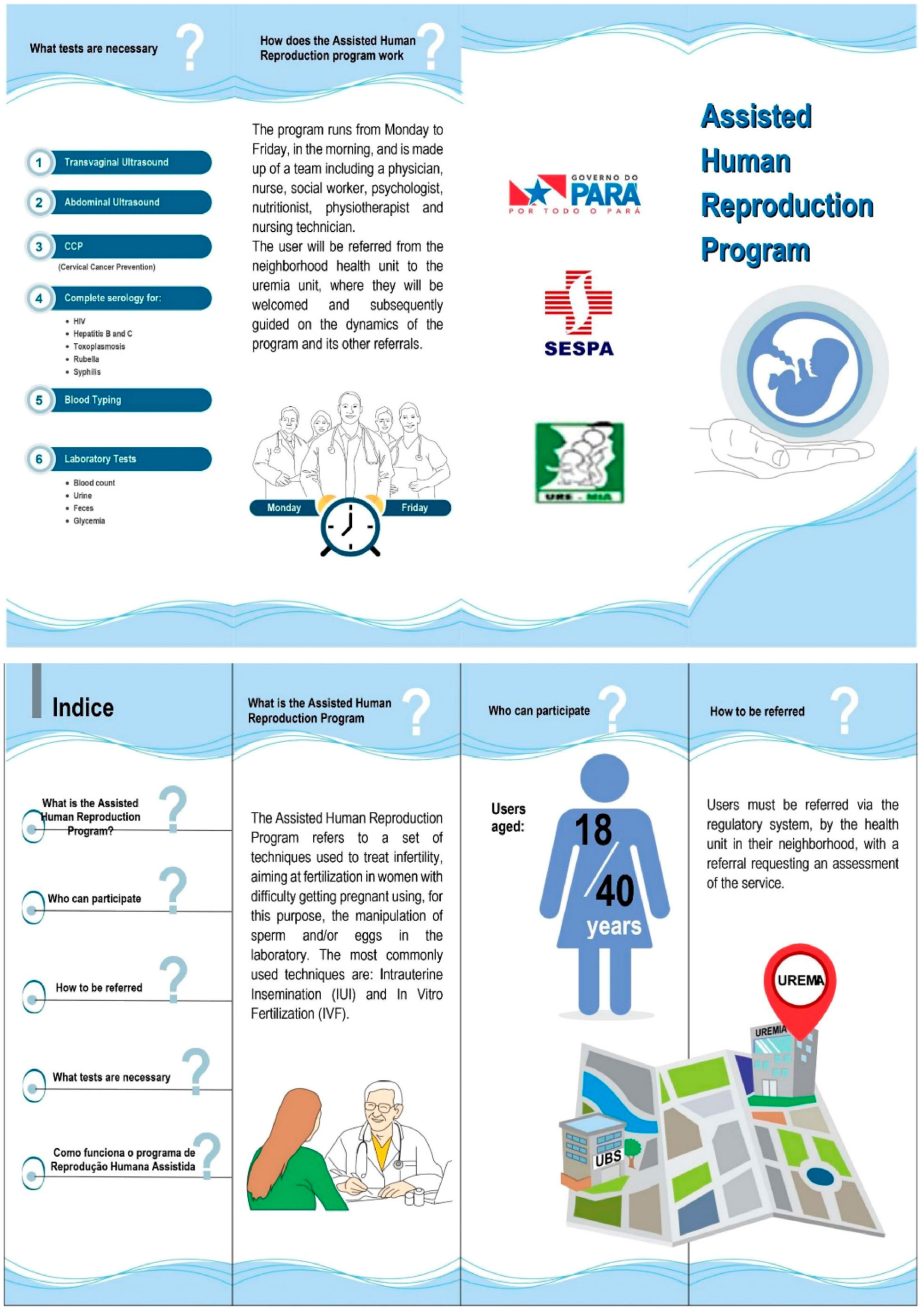
Folder on the Assisted Human Reproduction Program. Belém, Pará, Brazil, 2020.

Furthermore, the ideas in the folder were exemplified through colorful illustrations with a predominance of the color blue, due to its symbolism of trust and security, making it an ideal choice for topics that require additional reinforcement and seriousness, such as healthcare services. It is also a versatile color that allows for harmonious and attractive compositions, making the folder more visually interesting and welcoming for the reader. The illustrations are composed of images and drawings that allude to the information presented in the texts. For the final version, support was obtained from an IT professional and a layout professional, who followed Corel DRAW GraphicsSuite X6 program stages (Figure 3).

## DISCUSSION

The age of the couples interviewed in this study was within the age limit recommended by the Federal Council of Medicine, which recommends a maximum age of 50 for women to become pregnant through artificial insemination. The limit was proposed because of obstetric risk, congenital malformation and comorbidities (diabetes and hypertension), considering that, after the age of 50, risks of hypertension in pregnancy, diabetes and premature births increase^(15)^.

Another relevant fact regarding the age variable for assisted reproduction is in relation to egg donors’ age, which cannot exceed 35 years, which influences the egg quality and, consequently, its fertilization. In relation to cis men, it is recommended that they donate to sperm banks at an age under 50, since children of older fathers run a greater risk of developing health problems^(15)^.

In relation to couples’ marital status, given the social advance in the creation of new family arrangements, the demand for AHR methods by people in stable unions, as in this study, has increased, mainly due to the high rate of infertility. Thus, scientific advances have allowed for increased access to these services and the realization of people’s sexual and reproductive rights, regardless of their marital status^(16)^.

Low socioeconomic and educational levels were also a relevant finding in this study, and these variables may be factors that influence the success of counseling until follow-up in the program, since, at times, there is no prioritization of reproductive techniques, especially within the SUS, which, in this way, creates and maintains an economic barrier, excluding those who cannot afford to pay for AHR medications, procedures and services. Furthermore, the supremacy of AHR in the private sector deepens inequalities and vulnerabilities with the impossibility of access to these technologies^(5,17)^.

Furthermore, in relation to health literacy, especially of women seeking the assisted human reproduction program, it is an aspect that confronts the SUS principles of equity and comprehensiveness, since the intersection of social markers is an important barrier to access, with an exclusionary treatment of these women, since they sometimes present themselves as non-cisgender, low-income, with low education and non-white, reinforcing that this profile, historically, does not have easy access to healthcare services in Brazil, confronting the SUS principles of equity and comprehensiveness^(3)^.

Regarding religious factors, all couples in this study had some religion; in this regard, respect for the universality of access to health and treatment, with the State’s compliance with the Universal Declaration of Human Rights, not allowing religious perspectives to be a barrier to access to AHR services, is an important measure, since the notion of procreation has long been encompassed by erroneous conceptions and taboos, such as civil and religious unions between cisgender men and women^(17)^.

It is worth noting that the word “child” was the most cited in category I, which encompassed a unique meaning, as it expressed couples’ cognitive association with the assisted human reproduction program. The study showed that women associate the concept of family with feelings of love, affection, support and perpetuation of the species, associated with the idea of completeness to fill a gap and give continuity to the family, through the dimensions of an idealized family model based on kinship and blood ties^(18)^.

Concerning the other words that stood out, such as treat, assistance, advise, accompany, egg, disease, uterus, pregnancy, desire, want and husband, the limited knowledge that couples have about the Assisted Human Reproduction Program was revealed. From another perspective, they verbalized that the Assisted Human Reproduction Program “treats uterine disease”, “advises and accompanies during pregnancy” and “advises the couple/parents on the desire/want to have a child”.

In this context, the study on the profile of infertile couples undergoing the AHR technique concluded that knowledge related to infertility issues, its causes and services was scarce among the population and healthcare professionals^(19)^.

It is also revealed that one in six couples has difficulty becoming pregnant. The study showed that health conditions in both sexes that can lead to infertility are related to varicocele, vasectomy, genetic causes, hormonal changes, living with HIV or no apparent cause for cisgender men and endometriosis, tubal, ovulatory and uterine changes, immunological causes, and non-apparent causes for cisgender women^(20)^.

The reports also showed an intense desire to become pregnant, which is deposited in the reproduction program, even if it is “*in vitro*”, since couples see the service as a means of helping to resolve the difficulty of becoming pregnant. Given this context, assisted reproduction offers real possibilities for men and women who have difficulty becoming pregnant and having a child^(21)^.

The relationship between nurses and AHR is still incipient. Moreover, there is a lack of formal and protocol-based information on the subject since the nursing training process, which can be a dilemma, since nurses’ performance is based on scientific foundations using technologies^(5)^. In this context, it was also noted from the reports that couples are eager for information about how the Assisted Human Reproduction Program works, as well as about the tests, therapeutic itinerary, referrals and ways of accessing the program.

Therefore, it can be inferred that the suggestion that couples prepare a folder, such as ET, may be an important strategy, since the folder is a textual genre that awakens interest in the language, since, when used in accessible language, it promotes effective communication between people with flexibility and versatility both in the production and presentation of a topic, in addition to allowing the use of languages and images adapted to the target audience, which facilitates the rapid understanding of the ideas and concepts addressed in this work^(22,23)^.

Studies show that ETs are valid instruments for mediating caring actions in different contexts that relate to the target audience’s characteristics and demands, through the use of senses, perceptions, emotions, speech, social and cultural contexts, supporting the construction of a message that demonstrates its true meaning^(24,25)^.

One of the limitations of this study is the choice of informants from a single AHR program. Therefore, further studies are suggested including couples enrolled in other programs, in order to explore other conditions.

The results of this study reinforce the importance of support, both by healthcare professionals and, especially, by nurses, in AHR programs, as it is a possibility of listening, approaching and supporting couples, favoring broad and integrative assistance that elucidates the role of couples in the quality of the decision to be made.

## FINAL CONSIDERATIONS

Couple conditions indicated the need for informed welcoming, and the collection and application of social evidence favored the development of a context-based ET. It was concluded, therefore, that the process of building technologies based on social evidence, as the first stage of the technological product development cycle, is a strategy that can support nurses’ work process in the welcoming carried out in AHR programs, through continued validity of the instrument with the target audience.

The words that emerged supported the inference of couples’ insufficient knowledge, and information collection from the couples assisted in the program made it possible not only to define the content, but also to choose the type of ET. Text organization was guided by the words with the highest frequency and high degree of significance, being appropriate to the target audience’s language, culture and knowledge, based on both the context and scientific evidence.

## DATA AVAILABILITY

The entire dataset supporting the findings of this study has been made available on SciELO Data and can be accessed at: https://wp.scielo.org/wp-content/uploads/Guia_curadoria_pt.pdf
