# Supplementary material for: Welcoming couples into the Assisted Human Reproduction Program: developing educational technology based on the context
Source: Rev Esc Enferm USP. 2025 Jul 7;59:e20240263. doi: 10.1590/1980-220X-REEUSP-2024-0263en (PMC12258055; doi:10.1590/1980-220X-REEUSP-2024-0263en)
Supplement: Translation

# Translation: Acolhimento de casais no Programa de Reprodução Humana Assistida: desenvolvimento de tecnologia educativa baseado no contexto*

**DOI:** 10.1590/1980-220X-REEUSP-2024-0263pt

**Authors:** Alzinei Simor, Ivonete Vieira Pereira Peixoto, Mônica Custódia de Couto Abreu Pamplona, Marcia Helena Machado Nascimento, Fernando Conceição de Lima, Rubenilson Caldas Valois, Mary Elizabeth de Santana, Elizabeth Teixeira

**Affiliations:** 1 Universidade do Estado do Pará, Belém, PA, Brasil.

**Keywords:** Reprodução, Infertilidade, Enfermagem, Acolhimento, Tecnologia Educacional

## Abstract

**Objetivo:**

Descrever o processo de desenvolvimento de uma tecnologia educacional para enfermeiros com vistas à mediação do acolhimento de casais em reprodução humana assistida.

**Método:**

Pesquisa de desenvolvimento com interface metodológica, desenvolvida em duas fases: diagnóstico situacional no contexto; e desenvolvimento do produto. O diagnóstico foi conduzido por meio de entrevistas semiestruturadas, com 20 casais, em Belém, Pará, Brasil, de junho a setembro de 2019, e utilizou-se para apoiar a análise dos dados o *software* IRAMUTEQ®. A produção utilizou recursos textuais e imagéticos e o programa *Corel DRAW GraphicsSuite X6*.

**Resultados:**

Do corpus, emergiram seis classes, que foram organizadas em três categorias representativas do contexto. A partir do que emergiu, foi produzida uma tecnologia educativa do tipo *folder* para mediar o acolhimento dos casais pelo enfermeiro.

**Considerações finais:**

As condições dos casais indicaram a necessidade de um acolhimento informado e o levantamento, e a aplicação de evidências sociais favoreceu o desenvolvimento de uma tecnologia educativa baseada no contexto.

## INTRODUÇÃO

A infertilidade é definida clinicamente como a falha em alcançar uma gravidez após 12 meses ou mais de relações sexuais regulares desprotegidas. Este é um problema de saúde pública que está crescendo no mundo, com prevalência ao longo da vida entre 17% e 26%^(1)^. Tem-se ainda que a chance de um casal fértil engravidar é de, aproximadamente, 15% a 25% por mês, e após um ano de tentativas, essa taxa cumulativa será de, aproximadamente, 80%. Por isso, são necessárias intervenções médicas apropriadas e iniciar uma investigação sobre infertilidade^(2)^.

É imperativo que, para muitos casais, a vontade de engravidar é uma necessidade, mas que esbarra em condições de saúde importantes. Gestar e maternar se configuram como questões de saúde, e por isso deveria estar assegurado por meio de políticas públicas que garantissem pleno acesso aos serviços para tratamento da infertilidade e/ou reprodução humana assistida (RHA), superando as barreiras de acesso como uma questão de justiça reprodutiva^(3)^.

O enfermeiro, nesse cenário, desempenha um papel crucial para a integralidade do cuidado no Programa de Reprodução Humana Assistida e fertilidade, fornecendo apoio, acolhimento, esclarecendo instruções médicas e oferecendo orientações educacionais, especificamente durante o acolhimento, com especial atenção à educação dos pacientes, com enfoque no cuidado físico e psicológico, orientação sobre os procedimentos, promoção da qualidade do cuidado em saúde e pela sensibilidade às necessidades, e histórias dos casais/indivíduos^(4)^.

Com o progresso da área de RHA nos últimos tempos, a enfermagem ganhou destaque nesse cenário, garantindo, fundamentalmente, o acolhimento humanizado, além de atuar como principal articuladora de informações, conduções com a equipe multidisciplinar e na coordenação da equipe de programa de RHA, promovendo assistência direcionada às necessidades dos casais, orientando e preparando-os em todos os procedimentos como parte do acolhimento^(5)^.

No estado do Pará, o serviço para atendimento de infertilidade pelo Sistema Único de Saúde (SUS) é realizado na Unidade de Referência Materno Infantil (UREMIA), a qual acolhe casais inférteis de todas as classes sociais e gênero, tentando engravidar por um programa de reprodução assistida. Nesse serviço, não são utilizadas ferramentas educativas, como tecnologias educativas (TEs), durante o acolhimento de casais inférteis. Dessa forma, acredita-se que o elo entre TE e medicalização e a humanização/acolhimento valoriza o trabalho do enfermeiro em relação às biotecnologias reprodutivas^(5,6)^.

Sabe-se que a TE, como ferramenta, pode mediar o planejamento, a implementação e a avaliação do processo de ensino-aprendizagem aproveitado em encontros educativos, fortalecendo a construção de conhecimentos entre o educando e o educador^(7)^. Como estratégias para educação em saúde, as TEs contribuem para aumentar o acesso à informação referente ao adoecimento, diagnóstico, tratamento e acesso aos serviços de saúde, considerando o contexto social e familiar^(8)^. A TE pode ser considerada um recurso educacional e de cuidado significativo, e pode ser utilizada em atividades de educação em saúde^(7)^.

Portanto, acredita-se que investir no acolhimento e na produção da TE para essa clientela poderá reduzir os medos, a ansiedade e o impacto dos casais em relação à “vontade de engravidar”, bem como esclarecer dúvidas em relação à saúde reprodutiva, aos exames necessários, situar e sobre o funcionamento do Programa de Reprodução Humana Assistida^(9)^.

Dessa forma, o objetivo deste estudo é descrever o processo de desenvolvimento de uma tecnologia educacional para enfermeiros com vistas à mediação do acolhimento de casais em RHA.

## MÉTODO

### Desenho do Estudo

Pesquisa de desenvolvimento^(9)^ com interface metodológica^(10)^, desenvolvida em duas fases. A primeira fase (diagnóstico situacional no contexto da RHA) foi uma pesquisa com abordagem qualitativa, conforme as recomendações dos *COnsolidated criteria for REporting Qualitative research*. A segunda fase (desenvolvimento do produto)^(9)^ foi baseada no contexto^(11)^. A modalidade de produção baseada no contexto consistiu no levantamento de informações/considerações sobre o público-alvo e com o público-alvo (casais atendidos em programa de RHA), obtidas mediante produção de dados primários, possibilitando identificar temas significantes e, a partir deles, desenvolver a TE^(11)^. Ressalta-se que este estudo ainda não foi disponibilizado no serviço e os participantes ainda não puderam ter acesso a versão final; no entanto, este passará pelas etapas de projetos de continuidade^(12)^, com posterior avaliação com o público-alvo em estudos futuros.

Fase I - diagnóstico situacional no contexto da reprodução humana assistida

### Local

O estudo foi desenvolvido em uma UREMIA, referência estadual em RHA, vinculada ao SUS, localizada na cidade de Belém, Pará, Brasil.

### População e Critérios de Seleção

Participaram 20 casais inférteis que procuraram tratamento para RHA na UREMIA. Os critérios de inclusão foram casais com idades entre 18 e 40 anos, em acompanhamento na unidade de referência há pelo menos seis meses. O critério de exclusão foi ser casal em que a mulher era histerectomizada e/ou apresentasse doença de base em tratamento (miomatose uterina, endometriose). Realizou-se a abordagem dos participantes na sala de espera, antes da consulta com o enfermeiro, e nesse momento, foi feito convite para participar da pesquisa, conduzindo aqueles que aceitaram a uma sala reservada onde foram acolhidos pelo pesquisador e informados sobre os procedimentos a serem realizados.

### Definição da Amostra e Coleta de Dados

Os dados foram coletados entre junho e setembro de 2019. As entrevistas semiestruturadas foram conduzidas mediante roteiro construído com 22 questões sobre perfil sociodemográfico e condições dos casais. As perguntas foram relacionadas: ao conhecimento dos casais sobre o Programa de Reprodução Humana Assistida; aos motivos da procura pelo atendimento no programa; aos meios e formas para o programa ajudar os casais no acolhimento; às sugestões de conteúdo significativos para serem socializados em uma TE; e às sugestões sobre um formato interessante de TE para reunir os conteúdos com vistas a ajudar os casais inférteis em atendimento no programa.

### Análise E Tratamento Dos Dados

As entrevistas foram transcritas pelos pesquisadores e organizadas em *corpus* textual. Posteriormente, o *corpus* foi analisado utilizando as três etapas da análise de conteúdo do tipo temática, com apoio do *software Interface de R pour les Analyses Multidimensionnelles de Textes et de Questionnaires* (IRAMUTEQ^®^), segundo o método de Reinet^(13)^.

O IRAMUTEQ^®^ possibilita a identificação do contexto em que as palavras ocorrem, executando uma análise lexical do material textual e dividindo o texto em classes hierárquicas, que são identificadas a partir dos segmentos de textos (STs) que compartilharam o mesmo vocabulário, facilitando, assim, que o pesquisador conheça seu teor. A classe é definida como um agrupamento constituído por vários STs de vocabulário homogêneo^(14)^.

Optou-se pelo método da Classificação Hierárquica Descendente (CHD), no qual os textos foram classificados segundo os respectivos vocabulários, e o conjunto deles foi dividido pela frequência das formas reduzidas. Direcionou-se para a aquisição das classes de ST que apresentaram vocabulários similares entre si e diferentes dos STs das outras classes. Para a análise da CHD, foi necessário ter um percentual de aproveitamento de, no mínimo, 75% de ST pelo IRAMUTEQ^®^, considerando que se a retenção fosse menor que o proposto, o *corpus* não estava representativo para este tipo de análise ou que o conteúdo do *corpus* era muito diversificado, não permitindo hierarquizá-los^(14)^. Assim, após o processamento, as categorias geradas das classes da CHD foram submetidas ao processo de análise temática.

### Aspectos Éticos

O presente estudo seguiu os preceitos éticos de pesquisa envolvendo seres humanos, elencados na Resolução n^o^ 466/2012, com aprovação pelo Comitê de Ética em Pesquisa de instituição universitária pública do norte do país. O consentimento dos participantes foi obtido por meio do Termo de Consentimento Livre e Esclarecido, sob Parecer n^o^ 3.308.311. Para preservação do anonimato, o participante recebeu as letras “CI”, de casais infertilidade, seguidas pelo número correspondente à ordem de realização das entrevistas.

Fase II - Desenvolvimento propriamente dito da tecnologia educacional sobre acolhimento de casais na reprodução humana assistida.

## RESULTADOS

### Diagnóstico Situacional

As características dos 20 participantes apontaram que a idade variou entre 18 e 40 anos. Desses, 12 (60%) declararam união estável e oito (40%) estavam casados. Sobre o nível de escolaridade, 14 (70%) tinham o ensino médio completo e seis (30%) tinham o ensino médio incompleto. Além disso, 19 (95%) casais declararam renda familiar de um salário mínimo e um (5%) casal informou renda familiar de mais de um salário mínimo. Sobre a religião, 12 (60%) dos casais se consideravam católicos e oito (40%) se consideravam evangélicos.

O *corpus* textual apresentou 20 unidades de texto, 76 STs, 407 formas distintas e 2.667 ocorrências de palavras no texto, com frequência média das formas de 35.092.105, gerando seis classes semânticas distintas. Aproveitaram-se 62 STs de um total de 76, que corresponderam a 81,58% do *corpus*, caracterizando os STs. A CHD identificou a relação entre as classes e os vocábulos com maior associação nessas classes e seus respectivos qui-quadrados e frequências, tendo sido reportados apenas aqueles que atenderam ao critério estabelecido [χ^2^ (1) ≥ 3,84, p < 0,05], ou seja, as palavras significantes (Figura 1).

**Figura 1:** Classes e palavras com as respectivas frequências e qui-quadrado originadas do processo de análise dos dados do corpus textual “Tecnologia educativa para casais no Programa de Reprodução Humana Assistida”. Belém, Pará, Brasil, 2020.

Nota: *Grau de significância da palavra com a classe de <0,0001; ST - segmento de texto; f - frequência da classe; X2 - valor do qui-quadrado.

A partir disso, as categorias temáticas emergiram do agrupamento semântico 1, que são os conteúdos relacionados ao Programa de Reprodução Humana Assistida, e do agrupamento semântico 2, que são conteúdos relacionados à produção tecnológica, oriundos das seis classes geradas pelo IRAMUTEQ^®^, que exploraram lexicalmente o *corpus* textual “Tecnologia educativa para casais no Programa de Reprodução Humana Assistida”. As respectivas categorias temáticas foram interpretadas com base na análise de conteúdo proposta por Bardin^(12)^, organizadas em formato de fluxograma (Figura 2).

**Figura 2:** Categorias temáticas originadas do processo de análise dos dados do corpus textual “Tecnologia educativa para casais no Programa de Reprodução Humana Assistida”. Belém, Pará, Brasil, 2020.

#### Categoria 1: Conhecimentos Sobre o Programa de Reprodução Humana Assistida

Nessa categoria, no arranjo das classes VI e V, verificou-se que os vocábulos “espermatozoide” (n = 15) e “casal” (n = 10) foram os que mais se destacaram e os mais específicos, com alta significância. A palavra “filho” (n = 37), por sua vez, foi a mais citada, que englobou um sentido singular, por externar a associação cognitiva dos casais acerca do Programa de Reprodução Humana Assistida. Os vocábulos que se destacaram foram “tratar”, “assistência”, “aconselhar”, “acompanhar”, “óvulo”, “doença”, “útero”, “gravidez”, “desejo”, “querer” e “marido”, revelando o conhecimento limitado que os casais possuem sobre o Programa de Reprodução Humana Assistida. Os casais, assim, referiram:

*O Programa de Reprodução Humana Assistida faz orientações aos casais sobre como engravidar, podem pegar o meu óvulo e o espermatozoide do meu marido e implantar em mim e o programa fica acompanhando o processo. Achamos que é isso!* (CI-01)

*Na nossa opinião, o Programa de Reprodução Assistida vai aconselhar os casais que querem engravidar. Ele vai dizer quem pode, o que é preciso, se precisa fazer algum exame, enfim, acho que é isso!* (CI-03)

*O programa vai juntar meu óvulo e o espermatozoide do meu marido. Quero engravidar e ter um filho, para quando ficarmos mais velho ter uma pessoa para nos ajudar, descobrir a dificuldade que impede de engravidar, e os profissionais podem resolver a dificuldade que temos de engravidar de tratar essa doença*. (CI-06)

#### Categoria II: Motivos da Procura Pelo Programa de Reprodução Humana Assistida

Na categoria II, houve fusão das classes 2 e 3, verificando-se que os vocábulos “motivo” (n = 8) e “conseguir” (n = 9) foram o que mais se destacaram, ou seja, obtiveram alta significância nessa classe. As palavras “programa” (n = 44), “engravidar” (n = 41) e “filho” (n = 37) foram as mais citadas e apresentam valor não significante estatisticamente; no entanto, agregam relevante sentido para o contexto da categoria, por representar o principal motivo que levou os casais a procurar o serviço, pelo desejo de engravidar, ter um filho e descobrir a causa da dificuldade de engravidar. Os casais afirmaram:

*O principal motivo que nos fez procurar o programa foi para nos ajudar a engravidar e ter um filho, receber orientações sobre relação sexual, tratamento para engravidar e, por fim, colocar um óvulo e um espermatozoide no meu útero para engravidar*. (CI-17)

*O principal motivo é querer engravidar e ter um filho! Queremos ajuda para gerar um bebê. Um filho saudável! (CI-19)*

Os vocábulos seguintes que se destacaram foram “proveta”, “bebê”, “tratamento”, “doença”, “pensar”, “ajudar” e “sonho”, confirmando os motivos dos casais para buscar os serviços do Programa de Reprodução Humana Assistida. Assim, referiram:

*Realizar um sonho de gestar e um filho nosso, me dando as orientações e as informações necessárias para poder engravidar.* (CI-06)

*Como fazem bebê de proveta, queremos ter um. Não temos doença, penso que ele pode nos ajudar a engravidar e ter filhos e saber o porquê da dificuldade para engravidar. [...] o programa vai assistir a concepção de um filho. Procurei o programa para descobrirem a doença que nos impede de ter filhos. Queremos engravidar mesmo que seja de proveta!* (CI-09)

*O principal motivo é de eu ter um filho e engravidar, porque nunca engravidei! Para realizar este sonho, creio que o programa vai tratar essa doença e examinar os meus óvulos e os espermatozoides do marido.* (CI-15)

#### Categoria III: Necessidades e Sugestões Para Mediar o Acolhimento no Programa de Reprodução Humana Assistida

A categoria III surgiu da associação das palavras contidas nas classes 1 e 4, verificando-se que os vocábulos “*folder*” (n = 14) e “chegar” (n = 4) foram os que mais se destacaram, sendo mais específicos e alcançaram alta significância nessa classe. A palavra “exame” (n = 22) obteve grau de significância e foi citada nas duas classes, que englobou um sentido especial por externar a associação das dúvidas dos casais acerca dos exames necessários para ingressar no Programa de Reprodução Humana Assistida. Os casais, assim, mencionaram:

*Podia ter tipo um folder com informação com essa mensagem o que é o programa? Como faz para entrar? Que exames são necessários? Quem pode fazer é isso?* (CI-04)

*Que tenha informações de como chegar na unidade, do que é preciso para fazer parte, se precisa de exames? O que é o programa sobre a reprodução assistida? Tudo isso em um folder!* (CI-06)

*Um folder que tenha informações mais completas: como funciona o programa? O que é o programa? Quem pode participar? Como ser encaminhado para cá? Que exames precisa?* (CI-12)

Os vocábulos seguintes que se destacaram foram “necessário”, “explicar”, “informação”, “participar”, “encaminhar”, “funcionar” e “orientar”, desvelando as sugestões dos casais em relação aos conteúdos para compor um *folder*, que foi a TE de escolha para ser desenvolvida neste estudo.

Inferiu-se que os casais sugeriram um *folder* por ser claro e preciso, de fácil manuseio e leitura objetiva. Percebeu-se, ainda, pelos relatos, que os casais estavam ávidos por informações a respeito do funcionamento do Programa de Reprodução Humana Assistida, bem como dos exames, do itinerário terapêutico, dos encaminhamentos, do ingresso, entre outros, como observou-se nas falas:

*Pode ser um folder com mais informações, tipo explicando quem pode e quem não pode engravidar, quantos anos pode realizar reprodução assistida, se a pessoa é laqueada, poderá fazer parte desse programa. Explicar sobre os exames e os encaminhamentos!* (CI-01).

*Mais informações, como saber quem pode participar, os exames que precisam, como é o encaminhamento para cá. Essas coisas poderiam ter tudo num folder* (CI-08).

*Sugerimos um folder que tenha informações do que é o programa, como funciona, quem pode fazê-lo, como faz para chegar aqui, se precisa de exames. Um folder informando quem pode participar e quem não pode?* (CI-11).

## DESENVOLVIMENTO DA TECNOLOGIA EDUCATIVA

A partir dos temas que emergiram, chegou-se à modalidade *folder*, considerando o interesse dos participantes e os conteúdos necessários para o acolhimento. O *folder* foi elaborado em duas folhas tamanho A4 (21 cm x 29,7 cm), dividido em três partes, visando à possibilidade de ser dobrado, quando impresso, totalizando seis páginas, com orientação em paisagem. Utilizaram-se as fontes *Calibri* e *Times New Roman*, variando no tamanho da fonte, sendo 12 para o corpo do texto, 14, para os tópicos, e 20, para o título. O título “Programa de Reprodução Humana Assistida” contemplou o objetivo do *folder*.

O *folder* foi estruturado em cinco tópicos: “O que é o Programa de Reprodução Humana Assistida?”; “Quem pode participar?”; “Como é feito o encaminhamento ao programa?”; “Quais os exames necessários?”; “Como funciona o Programa de Reprodução Humana Assistida?”. Foram disponibilizadas as principais informações com demonstração objetiva da ação esperada, evitando o acumulo de orientações, com frases curtas, simples e uso da voz ativa.

Além disso, as ideias do *folder* foram exemplificadas por meio de ilustrações coloridas com predominância da cor azul, devido ao simbolismo de confiança e segurança, sendo uma escolha ideal para temas que precisam de reforço adicional e seriedade, como serviços de saúde, além de ser uma cor versátil e que permite composições harmoniosas e atraentes, tornando o *folder* visualmente mais interessante e acolhedora para o leitor. As ilustrações são compostas por imagens e desenhos que fazem alusão às informações apresentadas nos textos. Para a versão final, obteve-se apoio de um profissional da área da informática e de um da área da diagramação, que seguiram as etapas do programa *Corel DRAW GraphicsSuite X6* (Figura 3).

**Figura 3:** Folder sobre o Programa de Reprodução Humana Assistida. Belém, Pará, Brasil, 2020.

## DISCUSSÃO

A idade dos casais entrevistados neste estudo se encontrava dentro do limite de idade preconizado pelo Conselho Federal de Medicina, que recomenda a idade máxima de 50 anos para a mulher engravidar por inseminação artificial. O limite foi proposto por causa do risco obstétrico, malformação congênita e comorbidades (diabetes e hipertensão), considerando que, após os 50 anos, aumentam os ricos de hipertensão na gravidez, diabetes e partos prematuros^(15)^.

Outro dado relevante quanto à variável idade para a reprodução assistida é em relação à idade da doadora de óvulos que não pode ultrapassar os 35 anos, o que influencia a qualidade do óvulo e, consequentemente, a fecundação do mesmo. Já em relação aos homens cis, preconiza-se que doem com idade menor de 50 anos para bancos de esperma, já que filhos de pais mais velhos correm maior risco de desenvolver problemas de saúde^(15)^.

Sobre o estado civil dos casais, diante do avanço social na constituição de novos arranjos familiares, a procura por pessoas em união estável, como neste estudo, pelos métodos de RHA, tem aumentado, sobretudo, devido ao alto índice de infertilidade. Dessa forma, os avanços científicos permitiram ampliar o acesso a esses serviços e efetivar os direitos sexuais e reprodutivos das pessoas, independentemente do estado civil^(16)^.

Os baixos níveis socioeconômicos e de escolaridade também foram um achado relevante neste estudo, e essas variáveis podem se apresentar como fatores que influenciam o sucesso do aconselhamento até o acompanhamento no programa, visto que, por vezes, não há uma priorização das técnicas reprodutivas, sobretudo no âmbito do SUS que, dessa forma, cria e mantém uma barreira de ordem econômica, excluindo os que não podem pagar por medicamentos, procedimentos e serviços de RHA. Outrossim, a supremacia da RHA no setor privado aprofunda desigualdades e vulnerabilidades com a impossibilidade de acesso a essas tecnologias^(5,17)^.

Além disso, em relação à literacia em saúde, sobretudo de mulheres que buscam o programa de reprodução humana assistida, é uma vertente que confronta os princípios de equidade e integralidade do SUS, já que o entrecruzamento de marcadores sociais é uma importante barreira de acesso, com um tratamento excludente dessas mulheres, já que, por vezes, apresentam-se como não cisgênero, de baixa renda, com baixa escolaridade e não brancas, reforçando que esse perfil, historicamente, não dispõe de facilidade de acesso aos serviços de saúde no Brasil, confrontando os princípios da equidade e integralidade do SUS^(3)^.

Em relação aos fatores religiosos, todos os casais deste estudo apresentavam alguma religião; sobre isso, o respeito pela universalidade do acesso à saúde e ao tratamento, com o cumprimento pelo Estado da Declaração Universal dos Direitos Humanos, não permitindo que as perspectivas religiosas sejam uma barreira ao acesso aos serviços de RHA, é uma medida importante, já que a noção de procriação foi por muito tempo abarcada por concepções errôneas e tabus, como a união civil e religiosa entre homens e mulheres cisgêneros^(17)^.

Ressalta-se que a palavra “filho” foi a mais citada na categoria I, que englobou um sentido singular, por externar a associação cognitiva dos casais acerca do programa de reprodução humana assistida. Estudo evidenciou que mulheres associam o conceito de família a sentimentos de amor, carinho, sustentação e perpetuação da espécie, associado à ideia de completude para preencher uma lacuna e dar continuidade à família, mediante as dimensões de um modelo idealizado de família baseado em parentesco e laços consanguíneos^(18)^.

Em relação aos outros vocábulos que se destacaram, como tratar, assistência, aconselhar, acompanhar, óvulo, doença, útero, gravidez, desejo, querer e marido, revelaram-se os conhecimentos limitados que os casais possuem sobre o Programa de Reprodução Humana Assistida. Por outro olhar, verbalizaram que o Programa de Reprodução Humana Assistida “trata doença do útero”, “aconselha e acompanha durante a gravidez” e “aconselha o casal/pais no desejo/querer de ter filho”.

Nesse contexto, o estudo sobre o perfil dos casais inférteis submetidos à técnica de RHA concluiu que o conhecimento relacionado às questões da infertilidade, suas causas e serviços se mostrou escasso entre a população e profissionais de saúde^(19)^.

Revela-se ainda que, a cada seis casais, um apresenta dificuldade para engravidar. Estudo mostrou que as condições de saúde em ambos os sexos que podem levar à infertilidade estão relacionadas à varicocele, vasectomia, causas genéticas, alterações hormonais, conviver com HIV ou nenhuma causa aparente para homens cisgêneros e endometriose, alterações tubárias, ovulatórias e uterinas, causas imunológicas, e causas não aparentes para mulheres cirgêneros^(20)^.

Observou-se também, nos relatos, um intenso desejo de engravidar, que é depositado no programa de reprodução, mesmo que seja “*in vitro*”, uma vez que os casais enxergam o serviço como um meio de ajuda na resolução da dificuldade de engravidar. Diante desse contexto, a reprodução assistida oferece reais possibilidades de homens e mulheres com dificuldades de engravidar e gerar um filho^(21)^.

A relação do enfermeiro com a RHA ainda é incipiente. Além disso, há carência de informação protocolar e formal sobre o tema desde o processo de formação em enfermagem, o que pode ser um dilema, já que a atuação do enfermeiro está pautada no embasamento científico com utilização de tecnologias^(5)^. Nesse contexto, percebeu-se, ainda, pelos relatos, que os casais estão ávidos por informações a respeito do funcionamento do Programa de Reprodução Humana Assistida, bem como sobre os exames, o itinerário terapêutico, os encaminhamentos e as formas de acesso ao programa.

Por isso, infere-se que a sugestão de elaboração de um *folder* pelos casais, como TE, pode ser uma estratégia importante, já que o *folder* é um gênero textual que desperta o interesse pela língua, pois, ao ser utilizado com uma linguagem acessível, promove uma comunicação eficaz entre as pessoas com flexibilidade e versatilidade tanto na produção quanto na apresentação de um tema, além de permitir o uso de linguagens e imagens adaptadas ao público-alvo, o que facilita a compreensão rápida das ideias e dos conceitos abordados neste trabalho^(22,23)^.

Estudos demonstram que TEs são instrumentos validos para mediar o agir cuidativo em diferentes contextos que se relacionam às características e demandas do público ao qual se destina, por meio da utilização dos sentidos, percepções, emoções, falas, contextos sociais e culturais, fundamentando a construção de uma mensagem que demonstre seu verdadeiro significado^(24,25)^.

Uma das limitações do estudo é a escolha de informantes de um único programa de RHA. Desse modo, sugerem-se outros estudos incluindo casais matriculados em outros programas, a fim de explorar outras condições.

Os resultados deste estudo reforçam a importância do acolhimento, tanto por parte dos profissionais de saúde quanto por parte, especialmente, de enfermeiros, em programas de RHA, pois é uma possibilidade de escuta, aproximação e apoio aos casais, favorecendo uma assistência ampla e integradora que elucide o papel do casal na qualidade da decisão a ser tomada.

## CONSIDERAÇÕES FINAIS

As condições dos casais indicaram a necessidade de um acolhimento informado, e o levantamento e a aplicação de evidências sociais favoreceram o desenvolvimento de uma TE baseada no contexto. Concluiu-se, portanto, que o processo de construção de tecnologias pautadas em evidências sociais, como primeira etapa do ciclo de desenvolvimento de produtos tecnológicos, é uma estratégia que poderá subsidiar o processo de trabalho do enfermeiro no acolhimento realizado em programas de RHA, mediante a continuidade com validade do instrumento junto ao público-alvo.

Os vocábulos que emergiram sustentaram a inferência de um conhecimento insuficiente dos casais, e o levantamento de informações com os casais atendidos no programa possibilitou não só a definição dos conteúdos, mas também a escolha do tipo de TE. A organização do texto se guiou pelos vocábulos com maior frequência e alto grau de significância, sendo adequada à linguagem, cultura e saberes do público-alvo, baseando-se tanto no contexto quanto nas evidências científicas.

## DISPONIBILIDADE DE DADOS

Todo o conjunto de dados que dá suporte aos resultados deste estudo foi disponibilizado no SciELO Data e pode ser acessado em: https://wp.scielo.org/wp-content/uploads/Guia_curadoria_pt.pdf

# Translation: Acogida de parejas en el Programa de Reproducción Humana Asistida: desarrollo de tecnología educativa basada en el contexto*

**DOI:** 10.1590/1980-220X-REEUSP-2024-0263es

**Authors:** Alzinei Simor, Ivonete Vieira Pereira Peixoto, Mônica Custódia de Couto Abreu Pamplona, Marcia Helena Machado Nascimento, Fernando Conceição de Lima, Rubenilson Caldas Valois, Mary Elizabeth de Santana, Elizabeth Teixeira

**Affiliations:** 1 Universidade do Estado do Pará, Belém, PA, Brasil.

**Keywords:** Reproducción, Infertilidad, Enfermería, Acogimiento, Tecnología Educacional

## Abstract

**Objetivo:**

describir el proceso de desarrollo de una tecnología educativa para enfermeras con vistas a la mediación en la recepción de parejas en reproducción humana asistida.

**Método:**

investigación de desarrollo con interfaz metodológica, desarrollada en dos fases: diagnóstico situacional en el contexto; y desarrollo de productos. El diagnóstico se realizó a través de entrevistas semiestructuradas con 20 parejas en Belém, Pará, Brasil, de junio a septiembre de 2019, y se utilizó el software IRAMUTEQ® para apoyar el análisis de datos. La producción utilizó recursos textuales e imágenes y el programa Corel DRAW GraphicsSuite X6.

**Resultados:**

del corpus surgieron seis clases, las cuales se organizaron en tres categorías representativas del contexto. A partir de lo surgido se elaboró una tecnología educativa en formato de carpeta para mediar la recepción de las parejas por parte de la enfermera.

**Consideraciones finales:**

las condiciones de las parejas indicaron la necesidad de apoyo informado y la encuesta y la aplicación de evidencia social favorecieron el desarrollo de una tecnología educativa adaptada al contexto.

## INTRODUCCIÓN

La infertilidad se define clínicamente como la imposibilidad de lograr un embarazo tras 12 meses o más de relaciones sexuales regulares sin protección. Es un problema de salud pública creciente en todo el mundo, con una prevalencia a lo largo de la vida de entre el 17% y el 26%^(1)^. La probabilidad de que una pareja fértil se quede embarazada es aproximadamente del 15% al 25% al mes, y tras un año intentándolo, esta tasa acumulada será aproximadamente del 80%. Por ello, es necesario realizar las intervenciones médicas adecuadas e iniciar una investigación sobre la infertilidad^(2)^.

Para muchas parejas, el deseo de quedar embarazadas es una necesidad, pero choca con importantes condiciones en salud. El embarazo y la maternidad son cuestiones de salud y, por lo tanto, deben garantizarse a través de políticas públicas que aseguren el pleno acceso a los servicios de tratamiento de la infertilidad y/o reproducción humana asistida (RHA), superando las barreras de acceso como una cuestión de justicia reproductiva^(3)^.

En este escenario, las enfermeras desempeñan un papel crucial en la prestación de cuidados integrales en el programa de Reproducción Humana Asistida y fertilidad, proporcionando apoyo, acogida, aclarando las instrucciones médicas y ofreciendo orientación educativa, específicamente durante la acogida, con especial atención a la educación del paciente, centrándose en los cuidados físicos y psicológicos, la orientación sobre los procedimientos, la promoción de una atención sanitaria de calidad y la sensibilidad a las necesidades e historias de las parejas/individuos^(4)^.

Con los avances en el área de RHA en los últimos tiempos, la enfermería ha ganado protagonismo en este escenario, garantizando fundamentalmente una acogida humanizada, además de actuar como principal articulador de la información, orientación con el equipo multidisciplinar y coordinación del equipo del programa de RHA, promoviendo cuidados orientados a las necesidades de las parejas, orientándolas y preparándolas en todos los procedimientos como parte de la acogida^(5)^.

En el estado de Pará, la atención a la infertilidad es prestada por el Sistema Único de Salud (SUS) en la Unidad de Referencia Materno Infantil (UREMIA), que acoge a parejas infértiles de todas las clases sociales y géneros que intentan quedar embarazadas a través de un programa de reproducción asistida. En este servicio, no se utilizan herramientas educativas como las tecnologías educativas (TE) durante la acogida de las parejas infértiles. Por lo tanto, se cree que el vínculo entre las TE y la medicalización y la humanización/aceptación valora el trabajo de las enfermeras en relación con las biotecnologías reproductivas^(5,6)^.

Se sabe que la TE, como herramienta, puede mediar en la planificación, ejecución y evaluación del proceso de enseñanza-aprendizaje utilizado en los encuentros educativos, fortaleciendo la construcción del conocimiento entre el educando y el educador^(7)^. Como estrategias de educación en salud, las TE contribuyen a aumentar el acceso a la información sobre la enfermedad, el diagnóstico, el tratamiento y el acceso a los servicios de salud, teniendo en cuenta el contexto social y familiar^(8)^. Las TE pueden considerarse un importante recurso educativo y asistencial, y pueden utilizarse en actividades de educación en salud^(7)^.

Por lo tanto, se cree que invertir en la acogida de esta clientela y en la producción de TE puede reducir los miedos, la ansiedad y el impacto de las parejas en relación con el «deseo de quedarse embarazada», así como aclarar dudas sobre la salud reproductiva, las pruebas necesarias y el funcionamiento del Programa de Reproducción Humana Asistida^(9)^.

El objetivo de este estudio es describir el proceso de desarrollo de una tecnología educativa destinada a enfermeros para mediar en la acogida de parejas que se someten a RHA.

## MÉTODO

### Diseño del Estudio

Investigación para el desarrollo^(9)^ con una interfaz metodológica^(10)^, desarrollada en dos fases. La primera fase (diagnóstico situacional en el contexto del RHA) tuvo un enfoque de investigación cualitativa, en línea con las recomendaciones de los *COnsolidated criteria for REporting Qualitative research*. La segunda fase (desarrollo del producto)^(9)^ se basó en el contexto^(11)^. La modalidad de producción basada en el contexto consistió en la recogida de información/consideraciones sobre el público objetivo y con el público objetivo (parejas que asisten a un programa de RHA), obtenida mediante la producción de datos primarios, lo que permitió identificar temas significativos y, a partir de ellos, desarrollar la TE^(11)^. Cabe destacar que este estudio aún no está disponible en el servicio y que los participantes aún no pudieron acceder a la versión final; sin embargo, pasará por las etapas de los proyectos de continuidad^(12)^, con evaluación posterior con el público objetivo en futuros estudios.

Fase I - Diagnóstico situacional en el contexto de la reproducción humana asistida

Sitio del estudio

El estudio fue realizado en una UREMIA, centro estatal de referencia en RHA, vinculado al SUS, localizada en la ciudad de Belém, Pará, Brasil.

### Población y Criterios de Selección

Participaron 20 parejas infértiles que buscaron tratamiento para RHA en la UREMIA. Los criterios de inclusión fueron parejas con edad entre 18 y 40 años, con seguimiento en la unidad de referencia por al menos seis meses. Los criterios de exclusión fueron las parejas en las que la mujer había sido sometida a una histerectomía y/o tenía una enfermedad subyacente en tratamiento (miomatosis uterina, endometriosis). Las participantes fueron abordadas en la sala de espera antes de su cita con la enfermera, momento en el que se les invitó a participar en la investigación, y las que aceptaron fueron conducidas a una sala privada donde fueron recibidas por la investigadora e informadas de los procedimientos que se iban a llevar a cabo.

### Definición de la Muestra y Recolección de Datos

La Recolección de datos se realizó entre junio y septiembre de 2019. Las entrevistas semiestructuradas se realizaron mediante un guion de 22 preguntas sobre el perfil sociodemográfico y las condiciones de las parejas. Las preguntas estuvieron relacionadas con: el conocimiento de las parejas sobre el Programa de Reproducción Humana Asistida; las razones para solicitar atención en el programa; los medios y formas en que el programa puede ayudar a las parejas receptoras; sugerencias de contenidos significativos para socializar en una TE; y sugerencias de un formato de TE interesante para aglutinar los contenidos con miras a ayudar a las parejas infértiles que reciben atención en el programa.

### Análisis y Procesamiento de los Datos

Los investigadores transcribieron las entrevistas y las organizaron en un *corpus* textual. A continuación, el *corpus* se analizó mediante las tres etapas del análisis temático de contenido, con ayuda del *programa informático Interface de R pour les Analyses Multidimensionnelles de Textes et de Questionnaires* (IRAMUTEQ^®^), según el método de Reinet^(13)^.

IRAMUTEQ^®^ permite identificar el contexto en el que se producen las palabras, realizando un análisis léxico del material textual y dividiendo el texto en clases jerárquicas, que se identifican a partir de los segmentos textuales (ST) que compartían el mismo vocabulario, facilitando así al investigador el conocimiento de su contenido. Una clase se define como una agrupación formada por varios ST con vocabulario homogéneo^(14)^.

Optamos por el método de clasificación jerárquica descendente (CJD), en el que los textos se clasificaron según sus respectivos vocabularios, y el conjunto de textos se dividió por la frecuencia de las formas reducidas. Su objetivo era obtener las clases de ST que tenían vocabularios similares entre sí y diferentes de los ST de las demás clases. Para analizar la CJD, el IRAMUTEQ^®^ necesitaba utilizar al menos el 75% de los ST, considerando que si la retención era inferior a la propuesta, el *corpus* no sería representativo para este tipo de análisis o que el contenido del *corpus* era demasiado diverso, lo que hacía imposible clasificarlos^(14)^. Por lo tanto, tras el procesamiento, las categorías generadas a partir de las clases CJD se sometieron al proceso de análisis temático.

### Aspectos Éticos

Este estudio siguió los preceptos éticos de la investigación con seres humanos, establecidos en la Resolución 466/2012, con aprobación del Comité de Ética en Investigación de una universidad pública del norte del país. El consentimiento de los participantes fue obtenido a través del Formulario de Consentimiento Libre e Informado, bajo el Dictamen^nº^3.308.311. Para preservar el anonimato, los participantes recibieron las letras «CI», para parejas con infertilidad, seguidas del número correspondiente al orden en que se realizaron las entrevistas.

Fase II - Desarrollo real de la tecnología educativa sobre la acogida de parejas en reproducción humana asistida

## RESULTADOS

### Diagnóstico Situacional

Las características de los 20 participantes mostraron que sus edades oscilaban entre 18 y 40 años. De ellas, 12 (60%) declaraban una unión estable y ocho (40%) estaban casadas. En cuanto a su nivel de escolaridad, 14 (70%) tenían bachillerato completo y seis (30%) bachillerato incompleto. Además, 19 (95%) parejas declararon tener ingresos familiares de un salario mínimo y una (5A) pareja declaró tener ingresos familiares de más de un salario mínimo. Con relación a la religión, 12 (60%) de las parejas se consideraban católicas y ocho (40%) evangélicas.

El *corpus* textual presentó 20 unidades textuales, 76 STs, 407 formas distintas y 2.667 ocurrencias de palabras en el texto, con una frecuencia media de las formas de 35.092.105, generando seis clases semánticas distintas. Utilizamos 62 ST de un total de 76, que correspondían al 81,58% del *corpus*, caracterizando las ST. La CJD identificó la relación entre las clases y las palabras con mayor asociación en estas clases y sus respectivos chi-cuadrados y frecuencias, informando sólo de aquellas que cumplían el criterio establecido [χ^2^ (1) ≥ 3,84, p < 0,05], es decir, las palabras significativas (Figura 1).

**Figura 1:** Clases y palabras con sus respectivas frecuencias y chi-cuadrado del proceso de análisis de datos del corpus textual «Tecnología educativa para parejas en el Programa de Reproducción Humana Asistida». Belém, Pará, Brasil, 2020.

Nota: *Nivel de significación de la palabra con la clase de <0,0001; ST: segmento de texto; f: frecuencia de clase; X2: valor de chi-cuadrado.

De allí surgieron las categorías temáticas del agrupamiento semántico 1, que es contenido relacionado con el Programa de Reproducción Humana Asistida, y del agrupamiento semántico 2, que es contenido relacionado con la producción tecnológica, a partir de las seis clases generadas por el IRAMUTEQ^®^, que exploró léxicamente el corpustextual «Tecnología educativa para parejas en el Programa de Reproducción Humana Asistida». Las respectivas categorías temáticas fueron interpretadas a partir del análisis de contenido propuesto por Bardin^(12)^, organizado en formato de diagrama de flujo (Figura 2).

**Figura 2:** Categorías temáticas originadas en el proceso de análisis de datos del corpus textual «Tecnología educativa para parejas en el Programa de Reproducción Humana Asistida». Belém, Pará, Brasil, 2020.

#### Categoría 1: Conocimiento Sobre el Programa de Reproducción Humana Asistida

En esta categoría, en la disposición de las clases VI y V, las palabras «espermatozoide» (n = 15) y «pareja» (n = 10) fueron las que más se destacaron y las más específicas, con alto significado. La palabra «niño» (n = 37), por otro lado, fue la más citada, lo que engloba un significado singular, pues expresa la asociación cognitiva de las parejas con el Programa de Reproducción Humana Asistida. Las palabras que se destacaron fueron «tratar», «asistencia», «aconsejar», «acompañar», «óvulo», «enfermedad», «útero», «embarazo», «deseo», «querer» y «marido», revelando el conocimiento limitado de las parejas sobre el Programa de Reproducción Humana Asistida. Así informaron las parejas:

*El Programa de Reproducción Humana Asistida orienta a las parejas sobre cómo quedarse embarazadas, pueden coger mi óvulo y el esperma de mi marido e implantármelo y el programa hace un seguimiento del proceso. Creemos que ya está»* (CI-01)

*En nuestra opinión, el Programa de Reproducción Asistida asesorará a las parejas que quieran quedarse embarazadas. Les dirá quién puede, qué necesitan, si necesitan alguna prueba, en fin, ¡creo que ya está!»* (CI-03)

*El programa unirá mi óvulo y el esperma de mi marido. Quiero quedarme embarazada y tener un hijo, para que cuando seamos mayores tengamos a alguien que nos ayude, saber qué es lo que nos impide quedarnos embarazadas, y los profesionales puedan resolver la dificultad que tenemos para quedarnos embarazadas y tratar esta enfermedad.* (CI-06)

#### Categoría II: Motivos Para Solicitar el Programa de Reproducción Humana Asistida

En la categoría II, las clases 2 y 3 se fusionaron y las palabras «motivo» (n = 8) y «lograr» (n = 9) fueron las que más se destacaron, es decir, fueron altamente significativas en esta clase. Las palabras «programa» (n = 44), «quedar embarazada» (n = 41) e «hijo» (n = 37) fueron las más mencionadas y no fueron estadísticamente significativas; sin embargo, añaden un significado relevante al contexto de la categoría, ya que representan la razón principal que llevó a las parejas a buscar el servicio, debido a su deseo de quedar embarazada, tener un hijo y descubrir la causa de la dificultad para quedar embarazada. Las parejas dijeron

*El principal motivo por el que acudimos al programa fue para ayudarnos a quedarnos embarazados y tener un hijo, recibir orientación sobre las relaciones sexuales, tratamiento para quedarme embarazada y, por último, poner un óvulo y un espermatozoide en mi útero para quedarme embarazada.* (CI-17)

*La razón principal es que queremos quedarnos embarazadas y tener un hijo. Queremos ayuda para tener un hijo. ¡Un niño sano!*

Las siguientes palabras que destacaron fueron «probeta», «bebé», «tratamiento», «enfermedad», «pensar», «ayuda» y «sueño», confirmando las razones de las parejas para solicitar los servicios del Programa de Reproducción Humana Asistida. Así, mencionaron:

*Cumplir el sueño de quedar embarazada y tener un hijo propio, dándome la orientación e información necesarias para poder quedar embarazada* (CI-06).

Y*a que hacen bebés probeta, queremos tener uno. No tenemos ninguna enfermedad, creo que nos puede ayudar a quedarnos embarazadas y a tener hijos y a averiguar por qué es tan difícil quedarse embarazada. [...] el programa nos ayudará a concebir un hijo. He venido al programa para informarme sobre la enfermedad que nos impide tener hijos. Queremos quedar embarazados, ¡aunque sea de probeta*!» (CI-09)

*La razón principal es tener un hijo y quedarme embarazada, ¡porque nunca he estado embarazada! Para hacer realidad este sueño, creo que el programa tratará esta enfermedad y examinará mis óvulos y el esperma de mi marido.»* (CI-15)

#### Categoría III: Necesidades y Sugerencias Para Mediar la Acogida en el Programa de Reproducción Humana Asistida

La categoría III surgió de la asociación de las palabras contenidas en las clases 1 y 4. Las palabras «folleto» (n = 14) y «llegar» (n = 4) fueron las que más se destacaron, siendo más específicas y alcanzando alta significación en esta clase. Fue significativa la palabra «examen» (n = 22), mencionada en ambas clases, que tuvo un significado especial porque expresaba las dudas de las parejas sobre los exámenes necesarios para ingresar en el Programa de Reproducción Humana Asistida. Las parejas mencionaron lo siguiente

*Podría tener como un* folleto *con información con este mensaje ¿qué es el programa? ¿cómo inscribirse? ¿Qué exámenes se necesitan? ¿Quién puede hacerlo?* (CI-04)

*Debería haber información sobre cómo llegar a la unidad, qué hay que hacer para apuntarse, si hay que hacerse alguna prueba.... ¿Qué es el programa de reproducción asistida? Todo esto en un folleto* (CI-06)

*Un folleto con información más completa: ¿cómo funciona el programa? ¿En qué consiste el programa? ¿Quién puede participar? ¿Cómo ser derivado? ¿Qué pruebas necesita?* (CI-12)

Las siguientes palabras que se destacaron fueron «necesario», «explicar», «información», «participar», «derivar», «trabajar» y «orientar», revelando las sugerencias de las parejas sobre el contenido para componer un folleto, que fue la TE elegido para ser desarrollado en este estudio. Las parejas sugirieron un folleto porque era clara y precisa, fácil de usar y de leer. También quedó claro en los informes que las parejas estaban ávidas de información sobre el funcionamiento del Programa de Reproducción Humana Asistida, así como sobre las pruebas, el itinerario terapéutico, las derivaciones, la admisión, entre otros, según se observó en los discursos:

*Podría ser un folleto con más informaciones, como explicar quién puede quedar embarazada y quién no, qué edad se puede tener para la reproducción asistida, si la persona es lactante, puede formar parte de este programa. Explicar sobre los exámenes y las referencias a otros servicios* (CI-01).

*Más información, cómo saber quién puede participar, las pruebas que necesitan, cómo conseguirlas aquí. Todo esto podría estar en un folleto*(CI-08).

*Sugerimos un folleto con información sobre qué es el programa, cómo funciona, quién puede participar, cómo llegar, si necesita pruebas. Una folleto que diga quién puede participar y quién no* (CI-11).

## DESARROLLO DE LA TECNOLOGÍA EDUCATIVA

A partir de los temas que surgieron, ideamos la modalidad de folleto, teniendo en cuenta el interés de los participantes y el contenido necesario para darles la bienvenida. El folleto se elaboró en dos hojas de tamaño A4 (21 cm × 29,7 cm), divididas en tres partes para poder doblarlas al imprimirlas, con un total de seis páginas, en orientación horizontal. Se utilizaron los tipos de letra *Calibri* y *Times New Roman*, variando el tamaño de la fuente: 12 para el cuerpo del texto, 14 para los temas y 20 para el título. El título «Programa de Reproducción Humana Asistida» abarcaba el objetivo del folleto.

El folleto se estructuró en torno a cinco temas: «¿Qué es el Programa de Reproducción Humana Asistida?»; «¿Quién puede participar?»; «¿Cómo se realiza la derivación al programa?»; «¿Qué exámenes son necesarios?»; «¿Cómo funciona el Programa de Reproducción Humana Asistida?». La información principal se proporcionó con una demostración objetiva de la acción esperada, evitando la acumulación de directrices, con frases cortas y sencillas y el uso de la voz activa.

Además, las ideas del folleto fueron ejemplificadas a través de ilustraciones coloridas con predominio del color azul, por su simbolismo de confianza y seguridad, siendo una elección ideal para temas que necesitan refuerzo y seriedad adicional, como los servicios de salud, además de ser un color versátil que permite composiciones armoniosas y atractivas, haciendo el folleto visualmente más interesante y acogedor para el lector. Las ilustraciones se componen de imágenes y dibujos que aluden a la información presentada en los textos. Para la versión final se contó con el apoyo de un profesional informático y un maquetador, que siguieron los pasos del programa *Corel DRAW GraphicsSuite X6* (Figura 3).

**Figura 3:** Folleto sobre el Programa de Reproducción Humana Asistida. Belém, Pará, Brasil, 2020.

## DISCUSIÓN

La edad de las parejas entrevistadas en este estudio estaba dentro del límite de edad recomendado por el Consejo Federal de Medicina, que recomienda una edad máxima de 50 años para que la mujer quede embarazada por inseminación artificial. El límite fue propuesto debido al riesgo obstétrico, malformación congénita y comorbilidades (diabetes e hipertensión), considerando que después de los 50 años, aumenta el riesgo de hipertensión durante el embarazo, diabetes y partos prematuros^(15)^.

Otro aspecto relevante de la variable edad para la reproducción asistida es la edad de la donante de óvulos, que no puede tener más de 35 años, lo que influye en la calidad del óvulo y, consecuentemente, en su fecundación. En cuanto a los hombres cis, se recomienda que donen antes de los 50 años a los bancos de semen, ya que los hijos de padres mayores tienen más riesgo de desarrollar problemas de salud^(15)^.

En cuanto al estado civil de las parejas, dados los avances sociales en la creación de nuevas estructuras familiares, se ha incrementado la demanda de personas en uniones estables, como en este estudio, de métodos de RHA, especialmente debido a la alta tasa de infertilidad. Así, los avances científicos han permitido ampliar el acceso a estos servicios y hacer efectivos los derechos sexuales y reproductivos de las personas, independientemente de su estado civil^(16)^.

Los bajos niveles socioeconómicos y educacionales también fueron un hallazgo relevante en este estudio, y estas variables pueden ser vistas como factores que influyen en el éxito de la orientación y seguimiento en el programa, dado que a veces no hay priorización de las técnicas reproductivas, especialmente dentro del SUS, lo que crea y mantiene una barrera económica, excluyendo a aquellos que no pueden costear medicamentos, procedimientos y servicios de RHA. Además, la supremacía de la RHA en el sector privado profundiza las desigualdades y vulnerabilidades al imposibilitar el acceso a esas tecnologías^(5,17)^.

Además, en relación a la alfabetización en salud, especialmente entre las mujeres que buscan programas de reproducción humana asistida, este es un aspecto que confronta los principios de equidad e integralidad del SUS, ya que la intersección de marcadores sociales es una importante barrera de acceso, con un tratamiento excluyente de esas mujeres, pues a veces son presentadas como no-cisgénero, de baja renta, con baja escolaridad y no-blancas, reforzando que ese perfil históricamente no ha tenido fácil acceso a los servicios de salud en Brasil, confrontando los principios de equidad e integralidad del SUS^(3)^.

En cuanto a los factores religiosos, todas las parejas de este estudio tenían alguna forma de religión; en este sentido, el respeto al acceso universal a la salud y al tratamiento, con el Estado cumpliendo con la Declaración Universal de los Derechos Humanos y no permitiendo que las perspectivas religiosas sean una barrera para el acceso a los servicios de RHA, es una medida importante, ya que la noción de procreación ha estado cubierta durante mucho tiempo por conceptos erróneos y tabúes, como las uniones civiles y religiosas entre hombres y mujeres cisgénero^(17)^.

Cabe destacar que la palabra «hijo» fue la más citada en la categoría I, que engloba un significado singular, ya que expresa la asociación cognitiva de las parejas con el programa de reproducción humana asistida. Un estudio mostró que las mujeres asocian el concepto de familia a sentimientos de amor, afecto, apoyo y perpetuación de la especie, asociados a la idea de completitud para llenar un vacío y dar continuidad a la familia, a través de las dimensiones de un modelo idealizado de familia basado en el parentesco y en los lazos consanguíneos^(18)^.

Las otras palabras que se destacaron, como tratar, asistencia, aconsejar, acompañar, óvulo, enfermedad, útero, embarazo, deseo, querer y marido, revelaron el conocimiento limitado de las parejas sobre el Programa de Reproducción Humana Asistida. Por otro lado, verbalizaron que el Programa de Reproducción Humana Asistida «trata la enfermedad uterina», «aconseja y acompaña durante el embarazo» y «aconseja a la pareja/padres sobre el deseo/querencia de tener un hijo».

En este contexto, el estudio sobre el perfil de las parejas infértiles que se someten a RHA concluyó que el conocimiento de la infertilidad, sus causas y servicios era escaso entre la población y los profesionales sanitarios^(19)^.

También reveló que una de cada seis parejas tiene dificultades para quedar embarazada. Un estudio mostró que las condiciones de salud en ambos sexos que pueden llevar a la infertilidad están relacionadas con varicocele, vasectomía, causas genéticas, alteraciones hormonales, vivir con el VIH o causas no aparentes para los hombres cisgénero y endometriosis, alteraciones tubáricas, ovulatorias y uterinas, causas inmunológicas y causas no aparentes para las mujeres cisgénero^(20)^. Los informes también mostraron un intenso deseo de quedar embarazada, que se sitúa en el programa de reproducción, aunque sea «*in vitro*», ya que las parejas ven en el servicio un medio para ayudar a resolver la dificultad de quedar embarazadas. En este contexto, la reproducción asistida ofrece posibilidades reales a hombres y mujeres con dificultades para quedarse embarazados y tener un hijo^(21)^.

La relación de las enfermeras con la RHA es todavía incipiente. Además, hay una falta de protocolo e información formal sobre el tema desde el proceso de formación de enfermería, lo que puede ser un dilema, ya que el trabajo de las enfermeras se basa en un fundamento científico con el uso de tecnologías^(5)^. En este contexto, también quedó claro en los relatos que las parejas están ávidas de informaciones sobre el funcionamiento del Programa de Reproducción Humana Asistida, así como sobre los exámenes, el itinerario terapéutico, las derivaciones y las formas de acceso al programa.

Por lo tanto, se infiere que la sugerencia de que las parejas elaboren un folleto como TE puede ser una estrategia importante, ya que el folleto es un género textual que despierta interés por el lenguaje, pues, al ser utilizado con lenguaje accesible, promueve la comunicación efectiva entre las personas con flexibilidad y versatilidad tanto en la producción como en la presentación de un tema, además de permitir el uso de lenguaje e imágenes adaptadas al público objetivo, lo que facilita la rápida comprensión de las ideas y conceptos abordados en este trabajo^(22,23)^.

Estudios han demostrado que las TEs son herramientas válidas para mediar la atención en diferentes contextos que se relacionan con las características y demandas de la audiencia a la que se dirige, mediante el uso de los sentidos, percepciones, emociones, habla, contextos sociales y culturales, apoyando la construcción de un mensaje que demuestre su verdadero significado^(24,25)^.

Una de las limitaciones del estudio es la elección de informantes de un único programa de RHA. Por lo tanto, se sugieren otros estudios que incluyan a parejas inscritas en otros programas, con el fin de explorar otras condiciones.

Los resultados de este estudio refuerzan la importancia de la acogida, tanto por parte de los profesionales sanitarios como especialmente de enfermería, en los programas de RHA, ya que es una oportunidad para escuchar, acercarse y apoyar a las parejas, favoreciendo una atención integral e integradora que clarifique el papel de la pareja en la calidad de la decisión a tomar.

## CONSIDERACIONES FINALES

Las condiciones de las parejas indicaron la necesidad de una acogida informada, y el relevamiento y la aplicación de la evidencia social favorecieron el desarrollo de TE basadas en el contexto. Por lo tanto, se concluyó que el proceso de construcción de tecnologías basadas en la evidencia social, como primera etapa del ciclo de desarrollo de productos tecnológicos, es una estrategia que podría apoyar el proceso de trabajo de las enfermeras en la atención prestada en los programas de RHA, a través de la continuidad y validez del instrumento con el público objetivo.

Las palabras que surgieron apoyaron la inferencia de conocimiento insuficiente por parte de las parejas, y la recolección de información de las parejas atendidas en el programa posibilitó no sólo la definición del contenido, sino también la elección del tipo de TE. La organización del texto se orientó por las palabras con mayor frecuencia y alto grado de significación, siendo adecuada al idioma, cultura y conocimiento del público objetivo, basándose tanto en el contexto como en la evidencia científica.

## DISPONIBILIDAD DE DATOS

Todo el conjunto de datos que respalda los resultados de este estudio ha sido puesto a disposición en SciELO Data y se puede acceder en: https://wp.scielo.org/wp-content/uploads/Guia_curadoria_pt.pdf
